# Effects and Eradication of *Mycoplasma* Contamination on Patient-derived Colorectal Cancer Organoid Cultures

**DOI:** 10.1158/2767-9764.CRC-23-0109

**Published:** 2023-09-27

**Authors:** Divya L. Dayanidhi, Wylie K. Watlington, John B. Mantyh, Gabrielle Rupprecht, David S. Hsu

**Affiliations:** 1 Department of Medicine, Division of Medical Oncology, Duke University Medical Center, Durham, North Carolina.; 2 Center for Genomics and Computational Biology, Duke University, Durham, North Carolina.

## Abstract

Patient-derived organoids are a useful platform for identification and testing of novel precision oncology approaches. Patient-derived organoids are generated by direct culture of patient samples. However, prior to development into patient-derived organoids, these samples are often processed for clinical use, opening the potential for contamination by *Mycoplasma* and other microbes. While most microbes can be detected by visual inspection, *Mycoplasma* can go undetected and have substantial impacts on assay results. Given the increased use of patient-derived organoids, there is a growing need for a standardized protocol to detect and remove *Mycoplasma* from organoid models. In the current study, we report a procedure for *Mycoplasma* removal by passaging organoids through mice as patient-derived organoid xenografts. *In vivo* passage of patient-derived organoids followed by re-establishment was 100% effective at decontaminating colorectal patient-derived organoids (*n* = 9), based on testing with the Sigma LookOut Mycoplasma PCR Detection Kit. This process can serve as a method to re-establish contaminated patient-derived organoids, which represent precious models to study patient-specific genomic features and treatment responses.

**Significance:**

Organoids are valuable models of cancer. *Mycoplasma* contamination can alter organoid drug sensitivity, so there is a need for a standardized protocol to detect and remove *Mycoplasma* from organoids. We report a simple procedure for removing *Mycoplasma* from organoids via *in vivo* passaging through mice followed by re-establishment of organoids.

## Introduction

Patient-derived models of cancer, such as cell lines, organoids, and patient-derived xenografts (PDX) are increasingly being utilized as preclinical models to facilitate the identification and development of new therapeutics. Patient-derived organoids (PDO) can accurately model the biology of patient tumors, both at the phenotypic and genotypic levels (1–2). Furthermore, PDO have been used to perform high-throughput drug screens and correlate with patient therapeutic response, making them a useful platform for precision oncology approaches to identify novel therapies (1–2).

While organoid models have clear potential to aid in preclinical development of new therapies, these patient-derived models are also susceptible to contamination during clinical processing. One particularly difficult contaminant is *Mycoplasma*. *Mycoplasma* are the smallest bacteria that can self-replicate and be less than 1 µm in size (3–4). Because of their small size and antibiotic resistance (3), they are frequently found as contaminants in cell culture (3–4). *Mycoplasma* can negatively affect eukaryotic cells in numerous ways, from altering DNA, RNA, and protein levels to changes in cell growth and viability (3, 5–6). Proper aseptic techniques can prevent *Mycoplasma* contamination, but if there are no backup stocks for an infected line, eradicating *Mycoplasma* may be difficult. The most commonly-used method for eliminating *Mycoplasma* from cultures includes various antibiotics, such as quinolones and BM-Cyclin, as well as Plasmocin and Plasmocure (3, 7–9). While antibiotics may kill *Mycoplasma*, they can also be extremely stressful to cancer cells due to their mechanisms, such as inhibiting protein synthesis or inducing double-strand DNA breaks (3). Another concern is that *Mycoplasma* may develop resistance to antibiotic treatments, thereby making it more difficult to eliminate.

Detection and treatment of *Mycoplasma* have been extensively studied in a variety of cancer cell lines (3, 7–11). As researchers turn to other patient-derived models of cancer more representative of tumors in the human body, proper decontamination methods need to be established for these models as well. To date, no studies have extensively shown the effects of *Mycoplasma* contamination on PDOs or how they can be successfully treated. Thus, there is a growing need for a standardized method of removing *Mycoplasma* from organoid cultures without compromising cells of interest. Here, we demonstrate the effects of *Mycoplasma* in colorectal cancer PDO lines and establish a standard protocol for decontaminating *Mycoplasma* from organoid lines based on testing with the Sigma LookOut Mycoplasma PCR Detection Kit.

## Materials and Methods

### Establishment and Maintenance of PDOs

The study was conducted in accordance with the U.S. Common Rule. Colorectal cancer patient tissue samples were collected with written informed consent under a Duke Institutional Review Board–approved protocol (Pro00089222), obtained from either the NCI's Cooperative Human Tissue Network or through Duke University BioRepository & Precision Pathology Center and written consent was obtained from each subject. Tissue samples were cut into pieces approximately 2 mm^3^ with a sterile scalpel and mechanically digested in C-tubes with 10 mL of DMEM using a gentleMACS Dissociator (Miltyenyi Biotec). The three protocols on the gentleMACS Dissociator for digesting human tumors, h_tumor_01, h_tumor_02, and h_tumor_03, were each performed twice. Cells and tissue fragments were filtered through 70 µm filters and centrifuged at 500 × *g* for 5 minutes. Supernatants were aspirated and a total of 1.25 × 10^5^ cells were plated in 50 µL domes composed of 30% cell suspension in media and 70% Matrigel (Corning).

Colorectal cancer PDOs were maintained in colorectal cancer media, which consisted of DMEM F12 media supplemented with 10 mmol/L HEPES, 1X Glutamax, 100 U/mL Penicillin/Steptomycin, 500 nmol/L A83-01, 1X B27 without vitamin A, 50 ng/mL EGF, 10 nmol/L Gastrin-1, 1.25 mmol/L N-Acetylcysteine, 10 mmol/L Nicotinamide, 100 ng/mL Noggin, 100 µg/mL Primocin, 10 nmol/L Prostaglandin E2, 100 ng/mL R-Spondin 1, and 3 µmol/L SB20210. All PDO were maintained in a 37°C humidified incubator at 5% CO_2_. *Mycoplasma*-positive lines were maintained in an isolated incubator under the same conditions.

After PDO were incubated for at least 3 days, media was removed from plates and used for *Mycoplasma* testing with the LookOut Mycoplasma Detection Kit (Sigma-Aldrich) according to the manufacturer's protocol. PCR products were electrophoretically separated in 1% agarose gels containing SYBR Safe intercalating dye and imaged using a LI-COR Odyssey imaging system. This kit tests for the following 19 species: *M. laidlawii*, *M. arginini*, *M. bovis*, *M. falconis*, *M. fermentans*, *M. hyorhinis*, *M. opalescens*, *M. primatum*, *M. salivarium*, *M. timone*, *M. agalactiae*, *M. arthritidis*, *M. cloacale*, *M. faucium*, *M. hominis*, *M. hyosynoviae*, *M. orale*, *M. pulmonis*, and *M. spermatophilum*. This includes the following seven species that account for 95% of *Mycoplasma* contamination in culture: *M. arginine, M. fermentans, M. hyorhinis*, *M. orale*, *M. laidlawii*, *M. salivarium*, and *M. hominis* (12–14).

### Treatment of PDOs with Antibiotics

Media was removed from organoids and replaced with fresh media containing Plasmocin (Invivogen) according to manufacturer's protocols. Following treatment, organoids were tested for *Mycoplasma* as stated above.

### Elimination of *Mycoplasma* by Passaging Through Mice

A total of 2 × 10^6^ cells from each *Mycoplasma*-positive colorectal cancer organoid were subcutaneously injected into JAX NOD.CB17-PrkdcSCID-J mice. After the tumors grew to approximately 0.5 cm^3^, mice were euthanized following Duke Institutional Animal Care and Use Committee (IACUC)-approved protocols, and the tumor was harvested. Tumors were mechanically digested in C-tubes with 10 mL of DMEM using a gentleMACS Dissociator (Miltyenyi Biotec) and the m_impTumor_01.01 protocol was performed twice. Cells and tissue fragments were filtered through 70 µm filters and centrifuged at 500 × *g* for 5 minutes. The supernatants were aspirated, and a total of 1.25 × 10^5^ cells were plated in 50 µL domes composed of 30% cell suspension in media and 70% Matrigel (Corning). *In vivo* passaged colorectal cancer PDO were maintained in colorectal cancer media as described above. Once organoids grew, they were authenticated to be human cells.

### Drug Treatment Dose–response Curves

Stock solutions for oxaliplatin, SN38, and 5-fluorouracil (5-FU) were prepared at 10 mmol/L in PBS, DMSO, and PBS, respectively. Once the organoids were confluent, media was aspirated, and 1 mL of PBS was added to each well to detach Matrigel domes. Matrigel was centrifuged at 750 × *g* for 5 minutes. A total of 1 mL of TrypLE Express (Gibco) was used to dissolve Matrigel and break down organoids. These mixtures were incubated for 5 minutes, and TrypLE was neutralized by adding 5 mL of DMEM F12 media with 10% FBS and 1% penicillin/streptomycin. After centrifuging at 750 × *g* for 5 minutes, supernatants were aspirated. Cells were plated in 96-well plates in 5 µL domes at a concentration of 2 × 10^3^ cells per well. Organoids were allowed to recover for 2 days before addition of drugs.

To add drugs, media was aspirated from all wells and replaced with colorectal cancer media containing 2X of each component in the RealTime Glo MT Cell Viability Assay kit (RTG; Promega). Organoids were treated with each of the three compounds in a nine-point dilution series with a dilution factor of three starting from 1 mmol/L for oxaliplatin, 8 µmol/L for SN38, and 1 mmol/L for 5-FU, with five replicates per dose. Fluorescence was measured every day for 3 days using a Varioskan Lux plate reader (Thermo Fisher Scientific). Plates were imaged using an Incucyte S3 live cell imaging system. IC_50_ values were calculated using a nonlinear curve fit with the log (inhibitor) versus response (three parameters) function in GraphPad Prism (GraphPad Prism, RRID:SCR_002798).

### High-throughput Drug Screens and Growth Studies

The NCI Oncology panel of 147 FDA-approved drugs was provided in 96-well plates by the Duke Functional Genomics Core Facility and tested in triplicate. Once the PDO were confluent, media was aspirated, and 1 mL of PBS was added to each well to detach the Matrigel domes. Matrigel was centrifuged at 750 × *g* for 5 minutes. A total of 1 mL of TrypLE Express (Gibco) was used to dissolve Matrigel and dissociate organoids. These mixtures were incubated for 5 minutes and TrypLE was neutralized by adding 5 mL of DMEM F12 media with 10% FBS and 1% penicillin/streptomycin. After centrifuging at 750 × *g* for 5 minutes, supernatants were aspirated. PDO cell suspensions were used to make MicroOrganoSpheres (MOS) as described previously (15). A total of 100 MOS were plated per well. Cell viability was assessed using the Cell Titer-Glo luminescent Cell Viability Assay kit (Promega) after 72 hours. Plates were imaged using an Incucyte S3 live cell imaging system. Percent killing was calculated as follows: 100*[1 − (average CellTiterGlo_drug_/average CellTiterGlo_DMSO_)].

PDO cell suspension was also plated in triplicate 5 µL domes in 96-well plates at a concentration of 2 × 10^3^ cells per well. Plates were imaged every other day for 14 days using an Incucyte S3 live cell imaging system or ImageXpress Pico (Molecular Devices). Cell viability was assessed using the Cell Titer-Glo luminescent Cell Viability Assay kit (Promega).

### Data Availability

The data generated in this study are available upon request from the corresponding author.

## Results

### Effects of Plasmocin on *Mycoplasma* Clearance and Growth Rates in Colorectal Cancer PDO Cultures

**FIGURE 1:**
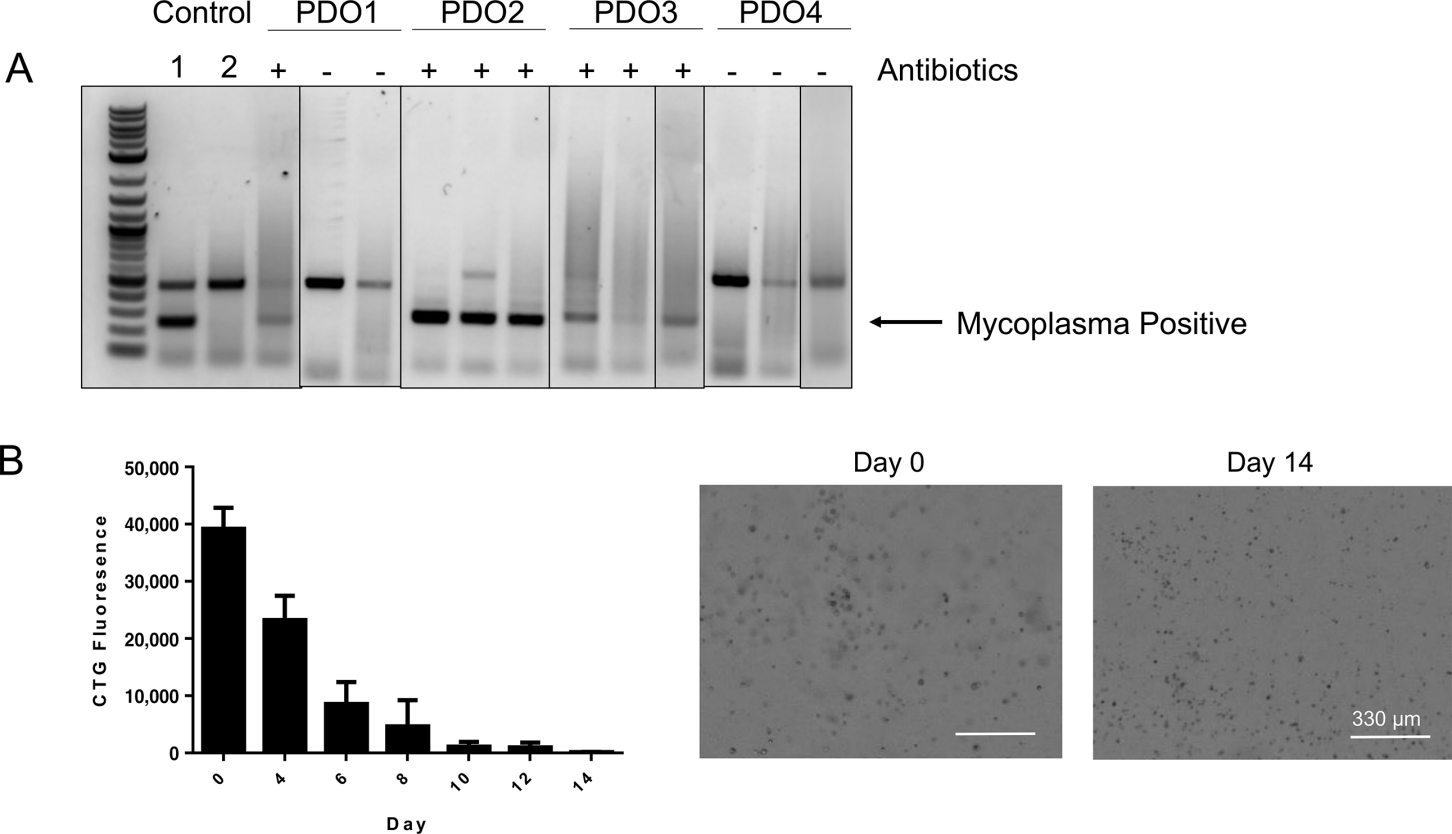
Plasmocin cannot reliably clear *Mycoplasma* from PDO lines. **A,***Mycoplasma* testing of *Mycoplasma*-positive lines treated with Plasmocin for 2 weeks according to the manufacturer's protocol. The top bands are a negative control for the PCR. The bottom bands are indicative of the presence of *Mycoplasma* (lanes 1 and 2). The top band may not appear if the original sample contained a high amount of *Mycoplasma*. All four lines were tested in triplicate. **B,** Growth rate of PDO4 after Plasmocin treatment over 14 days. Graphs on the left show CTG fluorescence of the PDO taken over the course of 14 days. Images on right compare PDO growth on days 0 and 14.

Several methods have been established to limit or remove *Mycoplasma* from cell cultures, including antibiotics and coculturing with macrophages. Currently, antibiotics remain the most common method for eliminating *Mycoplasma* from cultures (3, 7–8). To test the effectiveness of antibiotics on *Mycoplasma*-positive organoid cultures, we treated four patient-derived colorectal cancer PDO lines with Plasmocin in triplicate for 2 weeks according to the manufacturer's protocol. Treatment of organoids with Plasmocin had mixed results. In PDO1, two of three replicates converted to *Mycoplasma*-negative while PDO2 and PDO3 remained *Mycoplasma* positive (Fig. 1A), based on testing with the Sigma LookOut Mycoplasma PCR Detection Kit. Sample PDO4 was the only line that became *Mycoplasma* negative giving an overall conversion rate of 25% (1/4; Fig. 1A).

Because PDO4 was the only sample to be fully cleared by Plasmocin treatment, we evaluated the growth rate of PDO4 after treatment with Plasmocin by quantifying Cell Titer Glo fluorescence of the organoids over 2 weeks (Fig. 1B). The organoids produced the highest signal at the beginning and had a lower signal with every subsequent measurement, suggesting that Plasmocin negatively impacted the growth of PDO4 organoids (Fig. 1B).

### Converting *Mycoplasma*-positive PDOs to *Mycoplasma*-negative PDO by Passaging Through Mice

**FIGURE 2:**
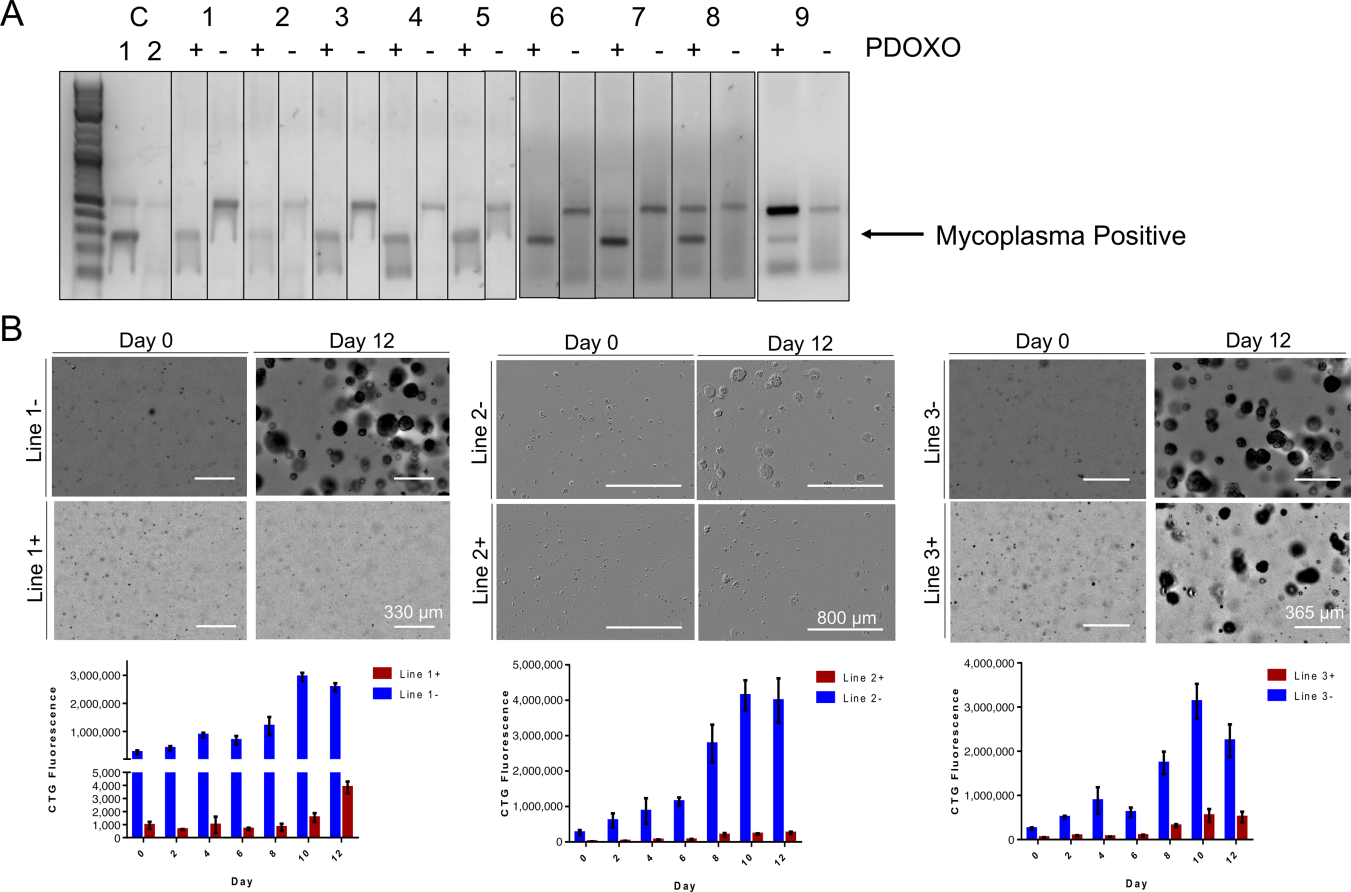
*Mycoplasma* can be successfully cleared from PDO lines by *in vivo* passaging through immunodeficient mice. **A,***Mycoplasma* test for organoid lines before (+) and after (−) *in vivo* passaging. Representative positive and negative control PCR products are included within lanes 1 and 2 of the gel on the left. The top band may not appear if the original sample contained a high amount of *Mycoplasma*. **B,** Growth comparison of *Mycoplasma*-positive and -negative lines over 12 days. Images on top compare PDO growth between positive and negative lines on days 0 and 12. Graphs on the bottom show CTG fluorescence of the PDO taken every other day for 12 days.

We next tested whether passaging organoids through mice may remove *Mycoplasma* without the need for antibiotic treatments. To do this, we passaged colorectal cancer PDO lines through immunodeficient mice (JAX NOC.CB17-PrkdcSCID-J mice) as patient-derived organoid xenografts. We tested this system on *n* = 9 *Mycoplasma*-positive colorectal cancer organoids by inoculated these cells subcutaneously into the flanks of JAX NOC.CB17-PrkdcSCID-J mice. Tumors were followed until they reach a size of approximately 0.5 cm^3^. Tumors were then harvested and regrown as organoids. All lines were authenticated after growing as organoids (Supplementary Fig. S1). Using this method, we achieved a 100% *Mycoplasma* clearance rate from the organoid lines based on testing with the Sigma LookOut Mycoplasma PCR Detection Kit (Fig. 2A). This kit tests for all seven *Mycoplasma* species that account for 95% of *Mycoplasma* contamination, in addition to 12 other species (12–14). On the basis of these results, we have formulated an easily-adoptable protocol for decontaminating *Mycoplasma*-positive colorectal cancer PDO as outlined in Table 1.

**TABLE 1:** Protocol for eliminating *Mycoplasma* contamination from PDO lines

Step	Instruction
1	Perform PCR for *Mycoplasma* on organoid lines using a commercially-available kit.
2	If lines test positive, isolate organoids onto a separate plate and move to a quarantine incubator.
3	Expand *Mycoplasma*-positive organoids to >2 × 10^6^ cells.
4	Inject 2 × 10^6^ cells per mouse subcutaneously into the flank of a JAX NOC.CB17-PrkdcSCID-J mouse.
5	Euthanize mouse following a standard IACUC-approved procedure and harvest the tumor when the size reaches approximately 0.5 cm^3^.
6	Digest the tumor as described in the Materials and Methods section and filter the solution into a single-cell suspension to replate as organoids.
7	Allow organoids to grow at least 3 days before retesting for *Mycoplasma*.
8	If organoids test positive for *Mycoplasma*, return to step 2. If lines test negative for *Mycoplasma*, remove organoids from quarantine incubator and return to original incubator.
9	Repeat *Mycoplasma* testing every 1–2 weeks to ensure no recontamination.

### *Mycoplasma* Contamination can Alter Organoid Growth and Drug Sensitivity

Quantification of growth rates of *Mycoplasma*-positive and -negative organoids by Cell TiterGlo indicated significant differences in matched *Mycoplasma*-negative and -positive lines, with CTG values at least five times higher in the *Mycoplasma*-negative lines (Fig. 2B). This suggests that *Mycoplasma* negatively impacts growth and viability of organoids and underscores the need to confirm *Mycoplasma* status in organoid models. Unlike eradication with antibiotics, passaging organoids through mice does not hinder the growth of the PDO.

**FIGURE 3:**
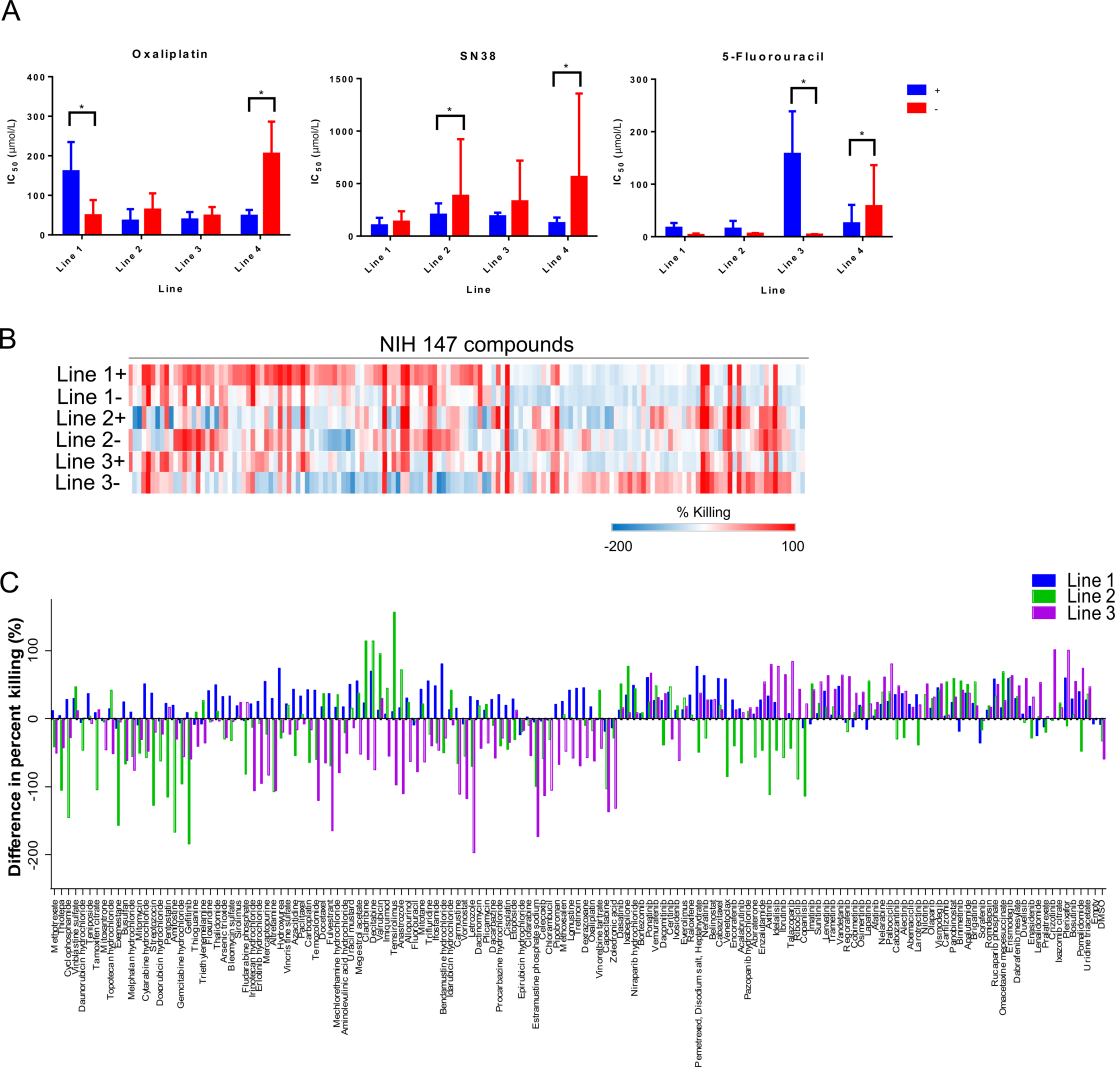
*Mycoplasma* contamination can change organoid drug sensitivity. **A,** Dose–response curves for four sets of *Mycoplasm*-positive and -negative lines for three drugs: oxaliplatin, SN38, and 5-FU. *, *P* < 0.05 (Mann–Whitney). **B,** High-throughput screens for three sets of *Mycoplasm*-positive and -negative organoid lines. Percent killing for each drug is indicated by color, with red being the highest percent killing and blue being the lowest. **C,** Difference in percent killing in high-throughput screen between *Mycoplasm*-negative and -positive organoid lines.

We next wished to determine whether *Mycoplasma* may impact organoid drug sensitivity. To do this, we performed dose–response curves for three specific standard-of-care drugs (oxaliplatin, irinotecan, and 5-FU) used in the treatment of colorectal cancer and found a significant difference in drug sensitivity between the *Mycoplasma*-positive and -negative colorectal cancer organoids (Fig. 3A). Specifically, in line 1, there was a difference in sensitivity to oxaliplatin (*P* < 0.05), but not to SN38 or 5-FU. Line 2 showed differences in sensitivity to SN38 (*P* < 0.05) only while line 3 showed differences in sensitivity to 5-FU (*P* < 0.05). Line 4 showed difference in all three drugs (*P* < 0.05; Supplementary Fig. S2).

We extended these analyses to high-throughput drug screens in three pairs of matched *Mycoplasma*-negative and -positive lines using a panel of 147 FDA-approved oncology agents. Analysis of these screens pinpointed both similarities and differences in drug sensitivity between pairs of *Mycoplasma*-negative and -positive lines (Fig. 3B). We plotted the relative drug sensitivity as a differential relative to the *Mycoplasma*-negative line for each matched organoid pair. These analyses revealed heterogeneous responses to drug depending on *Mycoplasma* contamination status (Fig. 3C; Supplementary Table S1).

Collectively, these results demonstrate that *Mycoplasma* contamination can substantially inhibit the growth of organoids and contributes to differential drug response in dose–response assays and high-throughput drug screens. These analyses highlight the importance of confirming the *Mycoplasma*-negative status of organoids prior to performing experiments.

## Discussion

*Mycoplasma* are common contaminants in cell cultures (4), and to date *Mycoplasma* detection and elimination has been extensively studied in cell lines (3, 7–11). PCR is a common detection method, and can be performed with commercial kits or custom-made primers. Unfortunately, *Mycoplasma* contamination can be difficult to eradicate as they can be less than 1 µm in size, allowing them to go through filters when most other bacteria would be eliminated (3). In addition, antibiotic treatments for cell lines, such as quinolones, BM-Cyclin, Plasmocin, and Plasmocure (3, 7–9), are not always effective against *Mycoplasma*. Many antibiotics target cell walls, but as *Mycoplasma* lack cell walls, they become difficult to eradicate (3). Thus, sterile techniques, filters with small mesh sizes, and autoclaving lab supplies are essential to prevent *Mycoplasma* contamination in cell culture. However, if *Mycoplasma* contamination does occur, it must be dealt with quickly and carefully to avoid further spread and contamination.

Accepted patient-derived models of cancer have expanded beyond cancer cell lines to three-dimensional (3D) models, including PDOs and PDXs. However, the effects of *Mycoplasma* on the growth or behavior of these patient-derived models have not been widely reported. One study by DesRochers and colleagues showed that 3D kidney tissue cultures contaminated with *Mycoplasma* were treated successfully with Plasmocin (6). With many researchers turning to organoids for cell culture, it is necessary to understand the effects of *Mycoplasma*, given many unwanted side effects in cell lines (3, 5–6).

*Mycoplasma* contamination has been known to negatively impact growth and viability of cancer cells (3, 6). We observed the same effect in multiple organoid lines (Fig. 2B). This inhibited growth may be due to cancer cells under extreme stress caused by multiple factors: competition for nutrients, physical invasion by *Mycoplasma*, and/or alterations to gene and protein expression or metabolism caused by *Mycoplasma* (3). Being under such conditions may result in a change in drug sensitivity for *Mycoplasma*-positive versus -negative cancer cells (16–17). We observed similar results, with *Mycoplasma*-positive organoids having lower IC_50_ values in general when compared with *Mycoplasma*-negative organoids of the same line (Fig. 3A). While this was not the case for every drug in our high-throughput screens, the vast majority had differing sensitivities between matched *Mycoplasma*-positive and -negative organoids (Fig. 3B and C).

Immunologic and chemotherapeutic methods have been used to eliminate *Mycoplasma* from cell lines (3). A few studies reported success in clearing *Mycoplasma* from cell lines by passaging through mice (18–20). We found that passaging organoid lines through mice has eliminated *Mycoplasma* successfully from 100% of the tumors that grew (Fig. 2A), based on testing with the Sigma LookOut Mycoplasma PCR Detection Kit. This kit tests for all seven *Mycoplasma* species that account for 95% of *Mycoplasma* contamination, in addition to 12 other species (12–14). This also provides an alternative method to antibiotics for those concerned about potential side effects on the cells of interest (3). While this method has advantages, there are also limitations. For one, organoids may not always grow as PDXs in mice, which could preclude the applicability of this method for some organoid models. In addition, there are almost certain to be influences on the heterogeneity and/or behavior of organoids subsequent to passaging through mice due to selective pressures inherent to the *in vivo* setting. Despite these limitations, however, we believe that this method provides a simple, reproducible, and complementary system to remove *Mycoplasma* from often-precious samples with advantages over traditional antibiotic treatments. Overall, our results indicate that standards must be adopted when researchers publish data using organoids. All organoid lines should be tested regularly for *Mycoplasma* before and after experiments and shown to be negative before publication to provide accurate and reliable data to the scientific community.

## Supplementary Material

Figure S1 Authentication of organoid lines after passaging through mice

Figure S2 IC50 curves for one round of standard of care drug treatment

Table S1 Table of average percent killing for all drugs in high throughput screens
