# Supplementary material for: Effects and Eradication of Mycoplasma Contamination on Patient-derived Colorectal Cancer Organoid Cultures
Source: Cancer Res Commun. 2023 Sep 27;3(9):1952–8. doi: 10.1158/2767-9764.CRC-23-0109 (PMC10530407; doi:10.1158/2767-9764.CRC-23-0109)

Supplementary Figure 1

A.

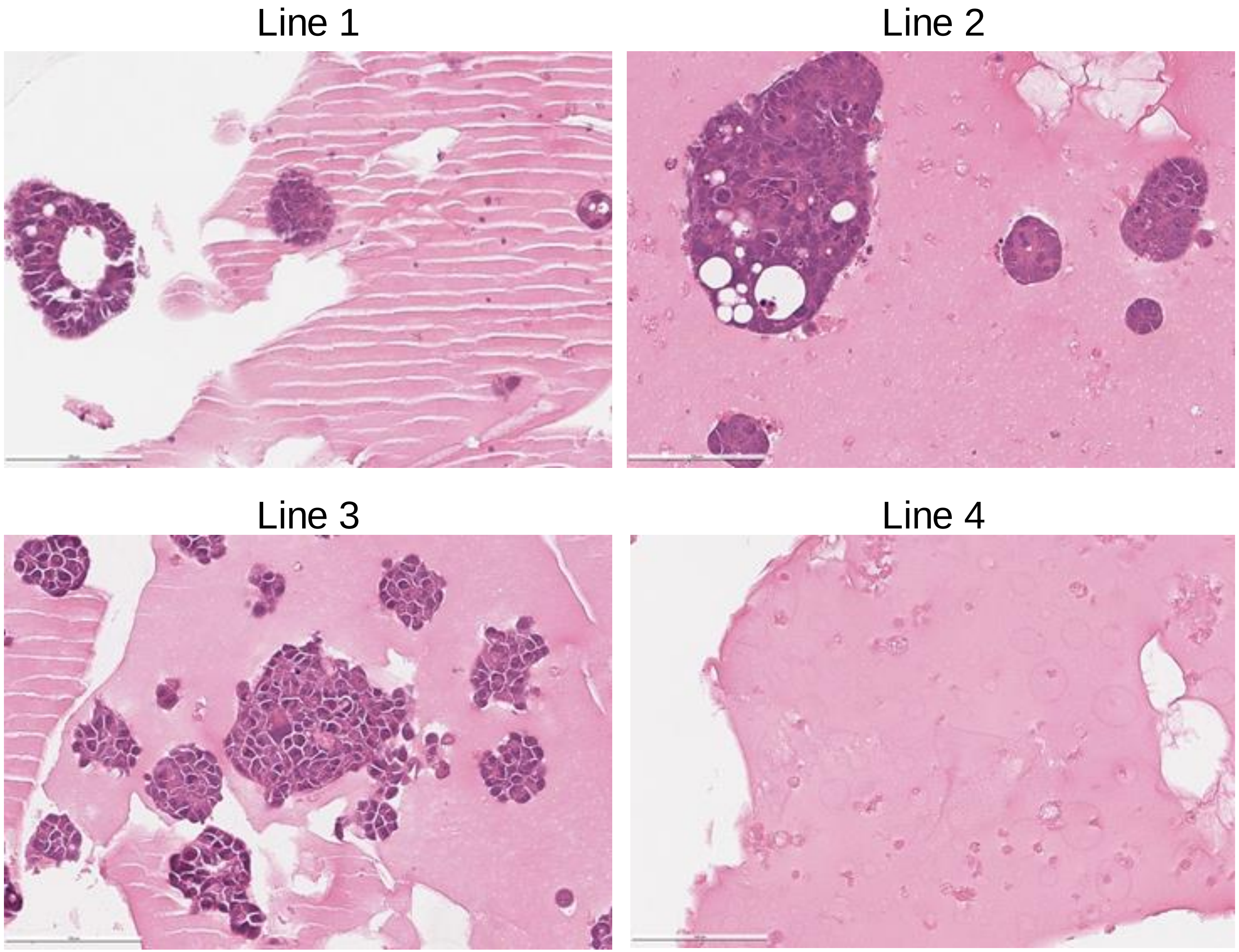

Supplementary Figure 1

B.

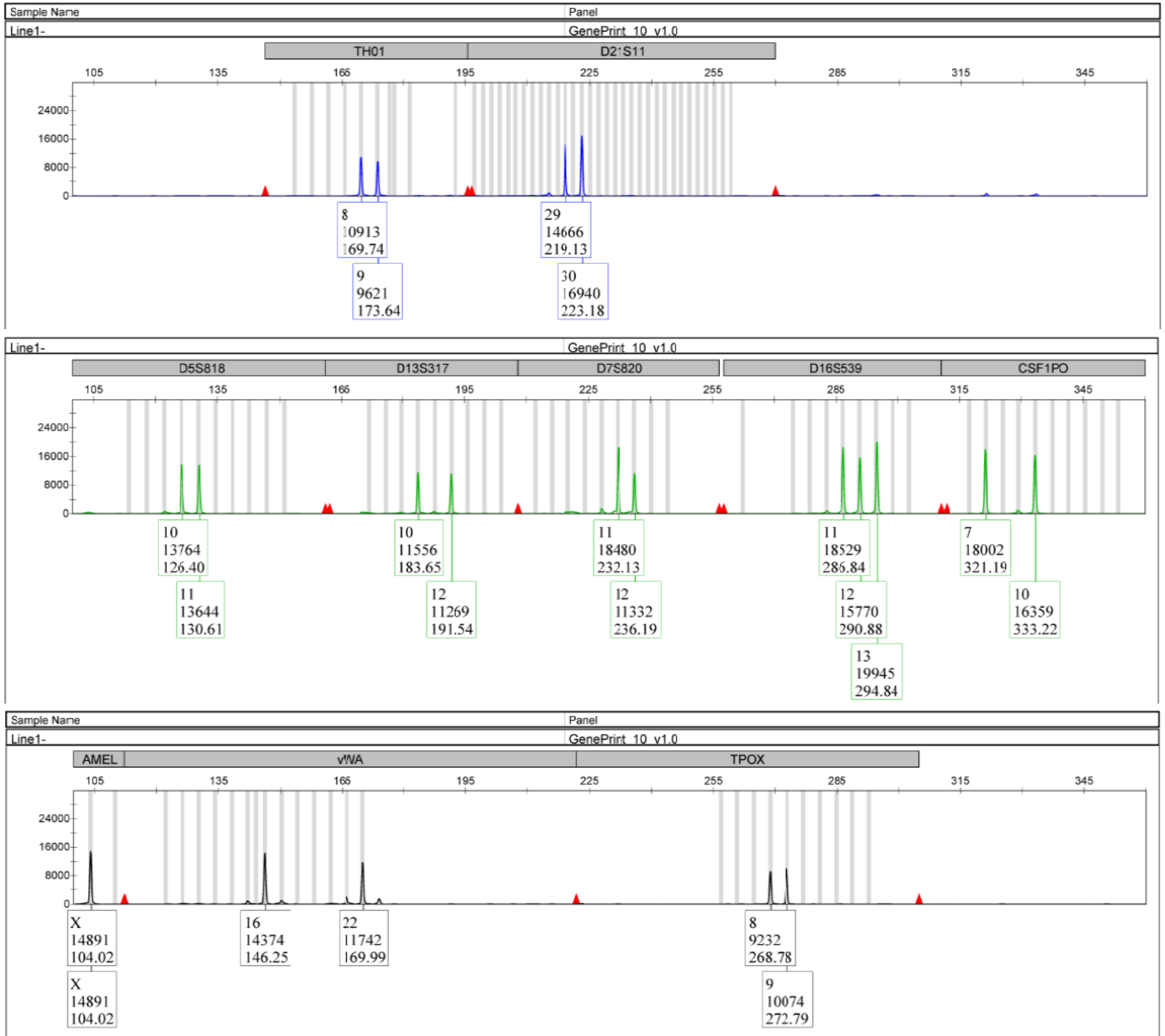

Supplementary Figure 1

B.

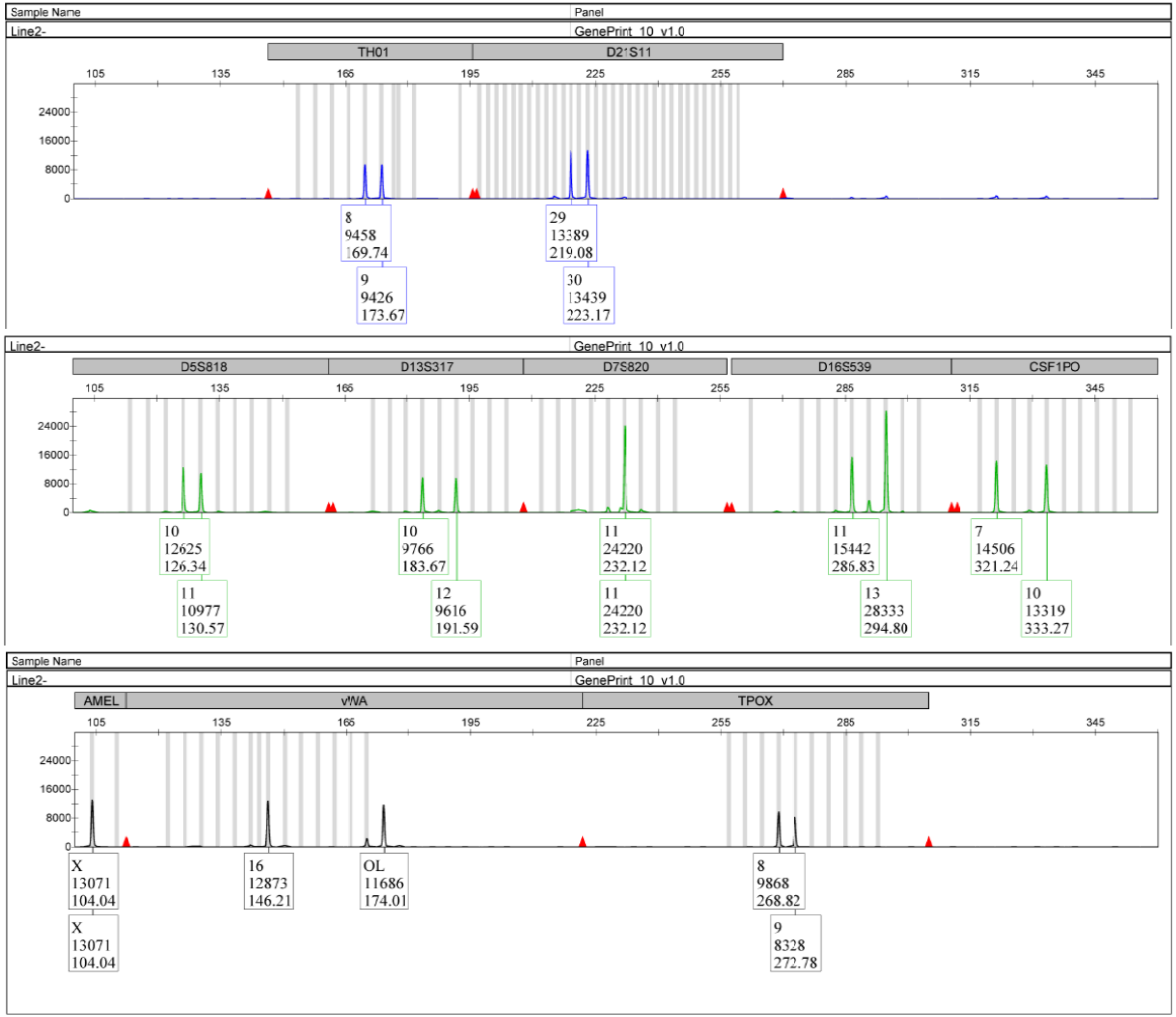

Supplementary Figure 1

B.

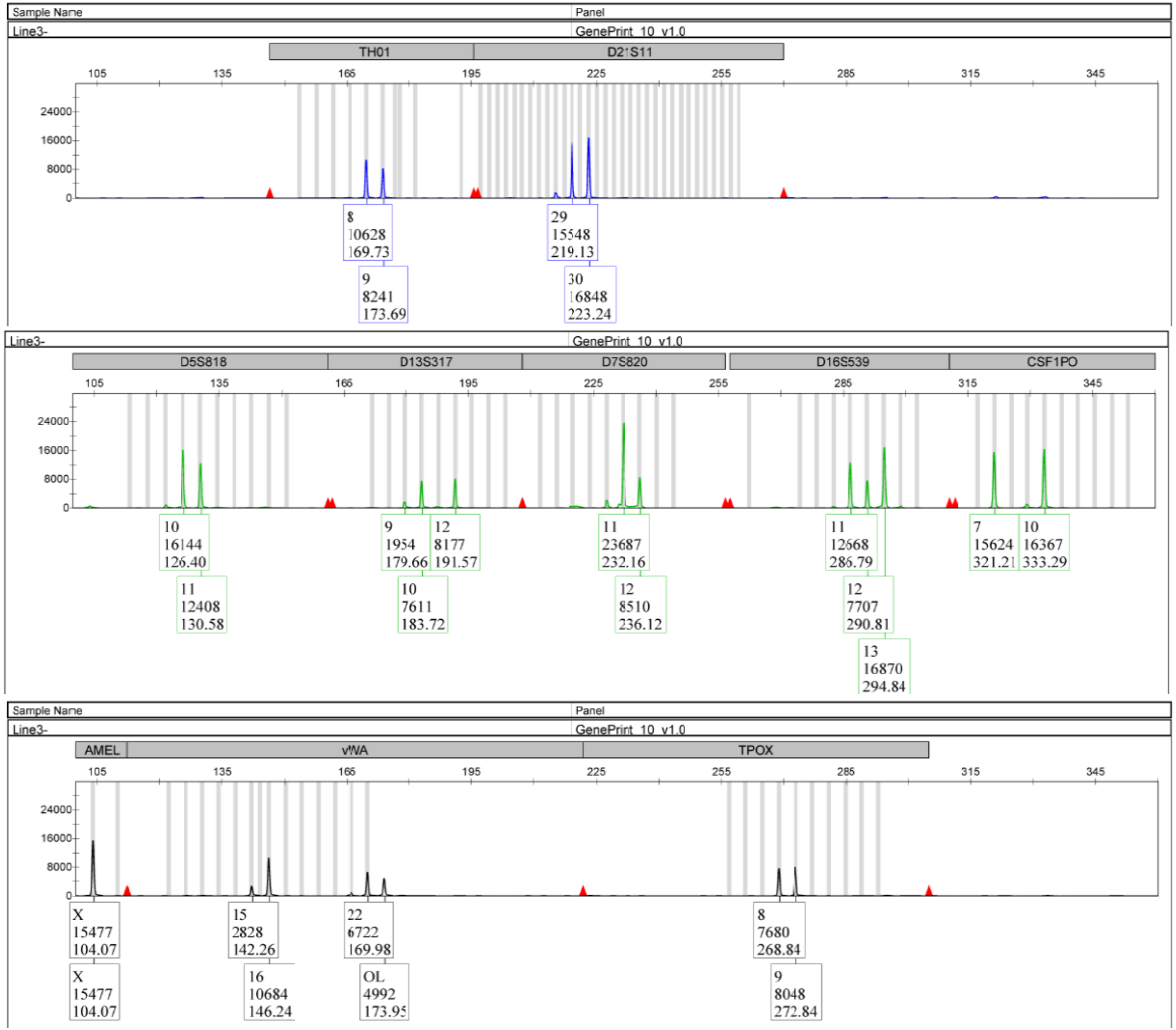

Supplementary Figure 1

B.

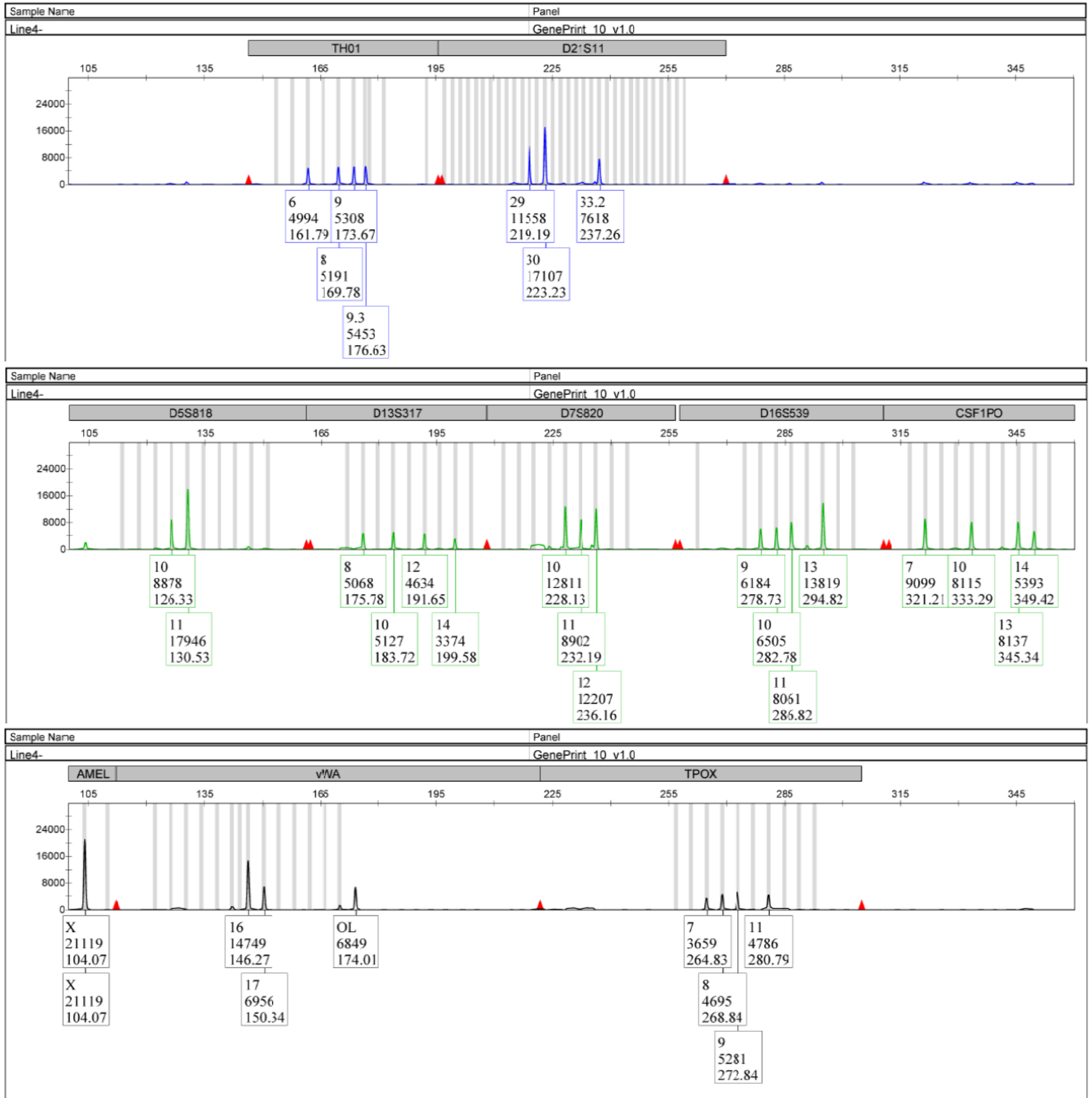

Supplement: Figure S1 — Authentication of organoid lines after passaging through mice [file crc-23-0109-s01.pdf]
