# Supplementary figures and images for: Effects and Eradication of Mycoplasma Contamination on Patient-derived Colorectal Cancer Organoid Cultures
Source: Cancer Res Commun. 2023 Sep 27;3(9):1952–8. doi: 10.1158/2767-9764.CRC-23-0109 (PMC10530407; doi:10.1158/2767-9764.CRC-23-0109)

# Supplementary Figure 2

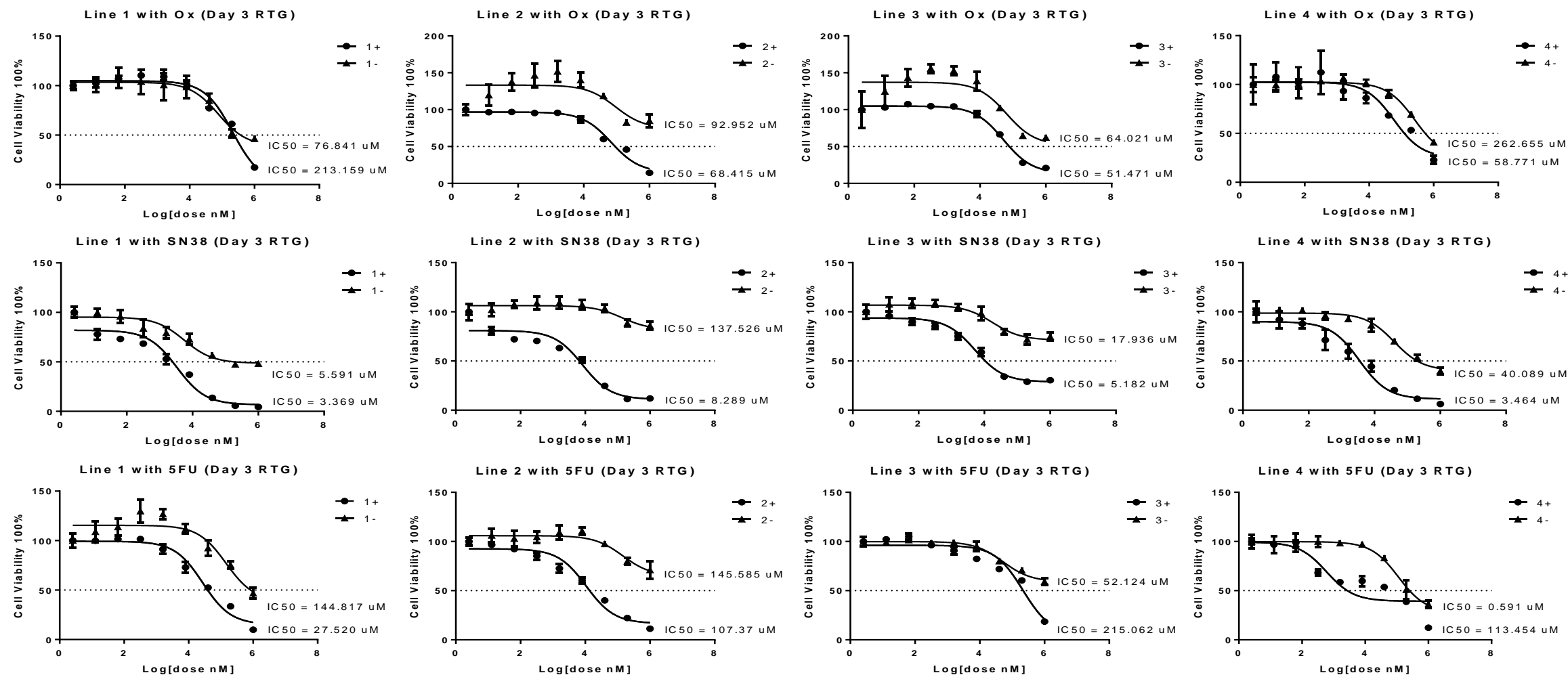

Supplement: Figure S2 — IC50 curves for one round of standard of care drug treatment [file crc-23-0109-s02.pdf]
