# Supplementary material for: Effects and Eradication of Mycoplasma Contamination on Patient-derived Colorectal Cancer Organoid Cultures
Source: Cancer Res Commun. 2023 Sep 27;3(9):1952–8. doi: 10.1158/2767-9764.CRC-23-0109 (PMC10530407; doi:10.1158/2767-9764.CRC-23-0109)
Supplement: Table S1 — Table of average percent killing for all drugs in high throughput screens [file crc-23-0109-s03.pdf]

Supplemental Table 1. The average percent killing values for matched mycoplasma-positive and –negative lines for each drug in the high throughput screen.

Drug	Line 1+	line 1-	Line 2+	line 2-	Line 3+	line 3-
Methotrexate	11.46383	56.59946	-41.5494	51.33387	-50.4958	68.70241
Thiotepa	4.41498	39.78999	-105.291	-43.0825	-42.8673	7.342794
Cyclophosphamide	27.70302	12.56896	-145.203	-38.4043	-27.9917	21.33481
Vinblastine sulfate	29.92407	60.07352	46.91694	14.90272	11.6885	54.60828
Daunorubicin hydrochloride	-6.03876	96.8853	-46.3964	25.98145	1.283106	93.87604
Teniposide	36.76888	41.96693	3.977644	-39.122	-7.14328	48.83125
Tamoxifen citrate	8.88641	36.48338	-104.673	-57.5157	13.41818	25.76395
Mitoxantrone	-2.59019	71.50311	-4.70475	-43.4546	-45.7584	74.08197
Topotecan hydrochloride	14.46133	77.20196	41.55271	31.85573	-51.4495	80.1439
Exemestane	-14.3572	43.72097	-156.56	-8.46148	-4.65946	40.48352
Busulfan	24.32004	31.60853	-66.5896	76.27962	-61.6602	18.42714
Melphalan hydrochloride	9.613343	53.45765	-55.8823	78.01585	-75.9367	38.35957
Mitomycin	-9.12362	87.17972	-50.4202	93.42054	-30.9131	71.70963
Cytarabine hydrochloride	50.96658	33.30968	-57.0848	83.70087	-47.5791	43.24823
Streptozocin	37.05782	38.43705	-127.548	65.19557	-19.9568	-5.39834

Doxorubicin hydrochloride	-3.29298	88.86014	-62.154	91.12054	-22.5855	92.85261
Pentostatin	22.02205	37.77785	-114.883	68.92959	16.42843	-0.60669
Amifostine	19.8428	47.01455	-167.024	33.85192	-29.7896	-3.71603
Gemcitabine hydrochloride	-6.25577	70.8705	-95.5934	77.65324	-55.7643	58.43751
Gefitinib	9.073951	36.43288	-184.239	54.90363	-59.1146	11.28435
Thioguanine	-8.81794	65.42416	9.362159	38.08087	-40.8266	65.07485
Triethylenemelamine	-7.66106	57.63875	26.81948	35.57167	-35.2774	20.83701
Floxuridine	40.8356	22.06615	-4.96191	4.464586	-3.35763	20.2907
Thalidomide	49.23764	25.57477	10.53527	-12.7968	-3.61678	-33.436
Arsenic trioxide	32.51622	42.30716	-29.642	-8.46414	-27.6436	8.532409
Bleomycin sulfate	32.83012	40.8346	-32.3113	33.23525	-4.23181	34.99751
Sirolimus	18.7369	62.16274	4.045849	3.159486	23.8796	24.73517
Fludarabine phosphate	-2.70428	96.30186	-81.2425	56.17125	23.85176	42.65853
Irinotecan hydrochloride	21.95927	44.76585	-12.7248	18.002	-105.976	53.97774
Erlotinib hydrochloride	25.33634	27.89258	-6.95747	9.918892	-95.273	56.83434
Mercaptopurine	54.81998	27.11909	-22.9153	66.86482	-82.9726	44.55272
Altretamine	29.2949	46.32878	-107.298	50.38229	-106.041	21.26843

Hydroxyurea	74.075	12.22873	-28.1868	32.13216	-19.7526	-13.7793
Vincristine sulfate	21.75816	72.71784	19.90771	59.38854	-22.7019	63.09386
Azacitidine	42.9593	42.53043	-54.3223	28.83353	-16.3165	7.434696
Paclitaxel	33.35115	62.61993	5.846864	61.38553	-7.30318	66.22107
Carboplatin	42.44041	32.61593	-64.1689	6.955173	-16.7936	16.76271
Temozolomide	41.79748	36.85817	-60.2659	5.477301	-120.14	43.04655
Docetaxel	16.90844	78.05678	36.65359	51.57576	-64.7534	83.4881
Fulvestrant	37.00128	37.12663	-69.5852	2.109559	-165.145	52.28011
Mechlorethamine hydrochloride	16.77829	32.64783	35.36397	-43.9118	-79.3509	24.31906
Aminolevulinic acid hydrochloride	17.44987	45.22303	-20.9218	3.672489	-50.9187	5.775362
Uracil mustard	50.48629	17.36939	15.12409	-33.8245	-13.7094	-2.69745
Megestrol acetate	55.36205	13.99899	37.15923	-59.4029	-52.0703	-2.21242
Cladribine	23.01145	46.25435	114.388	-92.7613	-60.0699	31.48227
Decitabine	69.83495	7.73398	114.0359	-102.756	-75.0911	24.10462
Valrubicin	12.07431	45.98589	95.76025	-98.2901	29.67007	-31.5214
Imiquimod	6.715192	59.71734	44.72775	-73.1981	-54.9206	-21.9483

Temsirolimus	9.937171	49.66196	156.8467	-133.988	-97.0473	7.143674
Anastrozole	16.07163	42.32842	71.84831	-69.9553	-110.086	-36.6015
Allopurinol	30.18965	-11.186	23.77201	16.80323	-62.792	17.76852
Fluorouracil	-8.88762	40.70436	-4.75666	28.6395	-78.0918	46.19674
Mitotane	43.32491	9.472341	21.82466	24.03311	-63.461	-5.80424
Trifluridine	55.39209	-0.73455	-22.857	18.14554	-40.1088	-10.0087
Ifosfamide	47.80387	19.68455	-36.0678	49.49982	-46.8495	-10.9916
Bendamustine hydrochloride	80.54692	-10.2424	-50.0374	30.14926	-28.695	1.79296
Idarubicin hydrochloride	13.15385	84.78031	41.33971	56.93171	-9.41526	95.05825
Carmustine	15.04433	38.00644	-65.9739	-11.6246	-111.398	17.18647
Vorinostat	-7.95565	62.10958	-55.0227	45.40644	-117.673	70.04264
Letrozole	32.67159	20.48196	-70.644	8.172557	-196.896	41.594
Dactinomycin	27.00118	64.76763	18.68809	80.40995	-43.6582	75.82478
Plicamycin	12.10218	86.15531	21.03074	78.65564	-35.3334	69.78481
Dacarbazine	28.16178	33.0359	-10.0936	28.21687	-57.6814	-3.00634
Procarbazine hydrochloride	35.33733	39.03245	-40.0071	45.66692	-28.239	-24.2458
Cisplatin	19.9182	50.09788	-45.2339	39.55342	-34.7546	-28.3303

Etoposide	28.99574	35.98899	-30.8118	59.28143	12.08063	-26.2625
Epirubicin hydrochloride	-23.0698	96.23435	-18.8085	89.62193	-17.3812	88.57357
Clofarabine	2.371442	76.08824	-9.80592	65.60757	-53.9398	25.20233
Estramustine phosphate sodium	-5.16267	47.84652	-99.7393	66.46346	-173.404	7.76401
Celecoxib	-3.57225	56.45858	-58.9035	54.01318	-113.428	-31.0491
Chlorambucil	-1.98583	23.19582	-30.584	64.9723	-105.012	34.19592
Pipobroman	20.82505	40.31097	-1.70508	44.48675	-67.4638	25.93499
Methoxsalen	25.36508	35.5318	5.778701	36.8688	-48.1249	13.2296
Lomustine	42.03375	30.60113	1.841482	18.86587	-57.8539	31.77585
Tretinoin	44.71189	30.52671	-1.31492	-4.5196	-68.959	14.87106
Dexrazoxane	45.08233	35.80292	-10.231	-41.089	-57.1824	10.16621
Oxaliplatin	17.1256	47.39731	-17.5015	-52.5186	-62.3831	20.88806
Vinorelbine tartrate	-1.48344	87.44526	41.55574	14.55452	-43.5673	76.23579
Capecitabine	-18.5435	52.98452	-103.491	-19.8858	-136.881	22.48103
Zoledronic acid	-13.6388	52.76656	-28.2162	-21.9856	-131.517	35.95737
Dasatinib	11.91897	-22.7485	50.87458	5.612861	16.07937	12.75733
Ixabepilone	34.58342	42.79092	77.03944	17.09561	8.965059	59.65776

Niraparib hydrochloride	48.72948	-26.2039	43.90195	-44.7571	9.059393	-3.79772
Bortezomib	7.782357	89.17485	9.143465	89.73915	-0.3037	93.35143
Ponatinib	60.26887	-30.8023	24.66168	-71.6831	66.8843	-17.9148
Vemurafenib	27.03696	-9.67089	47.94644	-41.089	30.96145	-20.9016
Dacomitinib	26.62334	1.91818	-38.296	4.791525	36.91758	-9.26076
Ceritinib	38.45854	-18.4956	46.56361	-37.0674	-29.9634	-14.3409
Ivosidenib	11.93472	4.972272	18.80862	-12.1748	-61.1935	-0.14974
Everolimus	13.39685	-7.6242	30.03686	23.57326	18.09147	30.68324
Raloxifene	34.47333	-34.0452	5.502783	43.53544	-3.01629	15.12124
Pemetrexed, Disodium salt, Heptahydrate	76.49089	-27.8253	-49.4827	54.69524	37.20468	4.925272
Neratinib	63.55714	-37.2082	-28.3622	44.18958	27.65843	-20.1868
Belinostat	27.58891	36.42491	1.881009	35.43824	27.77391	54.07474
Cabazitaxel	59.14369	18.51501	19.90244	67.08156	13.31823	54.70529
Venetoclax	58.43856	-19.1601	-84.827	36.4887	-0.12152	12.34632
Encorafenib	27.59546	6.338507	-40.0162	1.770924	13.47988	37.86433
Acalabrutinib	14.15634	-1.75789	-65.0167	-1.91233	8.817456	-2.95273
Pazopanib hydrochloride	-0.63858	-3.66105	10.60843	-12.3183	16.25846	5.469023

Abiraterone	6.895067	-12.8871	-56.465	26.63799	16.68089	9.796055
Enzalutamide	27.33482	-5.60408	-46.0056	54.85578	53.46668	-11.303
Axitinib	32.99458	-2.03433	-111.739	46.7833	80.04603	-36.8823
Idelalisib	23.63958	-12.2758	-46.4212	-6.18088	77.15436	-27.6155
Ibrutinib	-0.3145	-6.98627	-57.4691	-4.30164	64.24432	-34.0742
Talazoparib	7.625578	-3.50423	-43.7727	26.06119	84.24078	-39.4964
Ribociclib	-0.16877	17.11687	-89.0521	-12.4246	43.15359	-1.06109
Copanlisib	-13.9136	12.23139	-114.071	22.02602	21.67752	-4.38487
Imatinib	-7.52337	-1.72334	50.63011	-29.6594	42.67456	-14.5196
Sunitinib	7.776122	1.33341	22.02738	-24.7659	53.56361	3.758633
Lenvatinib	39.98664	-17.8311	17.47496	-28.3038	63.62536	-16.8171
Trametinib	5.278046	20.31716	17.09456	-0.02485	43.24226	28.67672
Vandetanib	47.22612	-20.5689	4.611908	-12.9908	63.77736	-20.7485
Regorafenib	-5.7572	17.90897	-19.5279	-16.7147	62.18965	-3.44032
Cobimetinib	-12.197	57.04601	9.191992	18.16946	37.14805	41.12428
Osimertinib	19.22817	6.038148	17.34412	-37.5006	38.78695	6.395697
Lapatinib	-15.4779	10.62327	55.10996	6.848851	21.77449	-2.8787

Afatinib	3.926395	13.43283	12.99055	29.68676	23.79524	20.08393
Nelarabine	20.93597	-5.26917	39.21277	22.43296	61.0515	-19.1912
Palbociclib	25.46831	7.252874	39.4155	-8.68476	80.32952	-34.7124
Cabozantinib	35.42278	-5.12032	-30.3337	52.25356	47.87503	-10.7005
Alectinib	36.5784	-4.53555	-27.9717	24.62691	40.66426	-13.0262
Abemaciclib	21.17236	16.85904	1.168553	20.84079	16.61701	15.23867
Larotrectinib	35.07661	5.421482	-38.8342	5.42414	20.29513	-7.57589
Nilotinib	-0.77946	-3.21184	35.09807	6.923276	50.92625	-9.26076
Olaparib	15.27243	-1.91472	29.37176	-0.82758	31.6173	-11.4817
Vismodegib	45.5482	-25.1673	42.22376	11.80876	51.10673	3.117875
Carfilzomib	3.384857	92.61569	53.80806	45.71716	4.734915	93.41474
Panobinostat	24.02266	72.45203	58.86056	40.78835	31.82489	62.36375
Binimetinib	-18.2671	29.80903	55.56031	-2.83972	41.5188	33.90235
Apalutamide	37.39538	-9.72405	50.59919	-18.2484	37.48255	13.47212
Brigatinib	22.38839	-6.4201	53.69833	-56.8841	18.78871	23.96677
Sorafenib	-35.9613	25.85121	-16.4464	70.54356	-2.4447	42.92657
Romidepsin	12.13622	84.0411	18.84341	80.82035	16.63083	77.77769

Rucaparib phosphate	57.99622	-23.4661	33.36599	11.53764	50.07405	-29.377
Omacetaxine mepesuccinate	15.49899	73.68802	68.78814	29.8303	26.44246	61.61833
Erismodegib	58.96666	-29.7125	60.6656	-7.89532	63.03094	-15.6683
Dabrafenib mesylate	29.52549	11.28778	32.4764	15.46357	48.47524	26.51704
Duvelisib	6.605397	2.024502	-5.40569	28.87606	59.12136	6.0817
Enasidenib	18.00205	-6.72046	-28.4462	43.16039	31.71291	8.787691
Lenalidomide	-24.6638	12.11444	2.768651	65.19823	52.99823	-10.2053
Pralatrexate	-12.1602	62.05908	-20.0092	88.02977	2.89945	68.0157
Crizotinib	-1.58798	27.21744	-2.64039	62.95431	101.5022	-33.1041
Ixazomib citrate	-0.70448	90.53763	22.85182	76.49226	15.7332	79.37576
Plerixafor	59.71989	-30.8289	-10.7956	52.97601	101.0384	-43.3154
Bosutinib	28.96617	2.473711	6.191239	33.61373	54.7816	1.154755
Pomalidomide	39.57809	-6.95969	-47.6505	35.03767	74.27476	-15.4131
Uridine triacetate	27.35712	5.549068	35.92736	-28.3543	46.5631	-25.0117
Empty	-7.70676	7.706758	6.66E-07	-3.6E-07	1.29E-07	-5.8E-07
DMSO	-8.56435	31.53626	-32.4069	-13.451	-59.0626	22.85469
